# Anti-angiogenic Therapy in Cancer: Downsides and New Pivots for Precision Medicine

**DOI:** 10.3389/fphar.2016.00519

**Published:** 2017-01-06

**Authors:** Gabriella Lupo, Nunzia Caporarello, Melania Olivieri, Martina Cristaldi, Carla Motta, Vincenzo Bramanti, Roberto Avola, Mario Salmeri, Ferdinando Nicoletti, Carmelina D. Anfuso

**Affiliations:** Department of Biomedical and Biotechnological Sciences, School of Medicine, University of CataniaCatania, Italy

**Keywords:** tumor angiogenesis, anti-angiogenic therapy, tumor endothelial cells (TECs), pericytes, vascular normalization, microvascular architecture, hypoxia detection

## Abstract

Primary solid tumors originate close to pre-existing tissue vasculature, initially growing along such tissue blood vessels, and this phenomenon is important for the metastatic potential which frequently occurs in highly vascularized tissues. Unfortunately, preclinic and clinic anti-angiogenic approaches have not been very successful, and multiple factors have been found to contribute to toxicity and tumor resistance. Moreover, tumors can highlight intrinsic or acquired resistances, or show adaptation to the VEGF-targeted therapies. Furthermore, different mechanisms of vascularization, activation of alternative signaling pathways, and increased tumor aggressiveness make this context even more complex. On the other hand, it has to be considered that the transitional restoration of normal, not fenestrated, microvessels allows the drug to reach the tumor and act with the maximum efficiency. However, these effects are time-limited and different, depending on the various types of cancer, and clearly define a specific “normalization window.” So, new horizons in the therapeutic approaches consist on the treatment of the tumor with pro- (instead of anti-) angiogenic therapies, which could strengthen a network of well-structured blood vessels that facilitate the transport of the drug.

## Cancer-Related Angiogenesis and Anti-Angiogenic Therapy

The blood vessels supplying tumors are permeable, tortuous, heterogeneous in their morphological structure and efficiency of perfusion, and greatly different from those composing the normal vasculature. These features determine what is now called “aberrant angiogenesis,” which characterizes the tumor environment (Huang et al., 2013).

**FIGURE 1:**
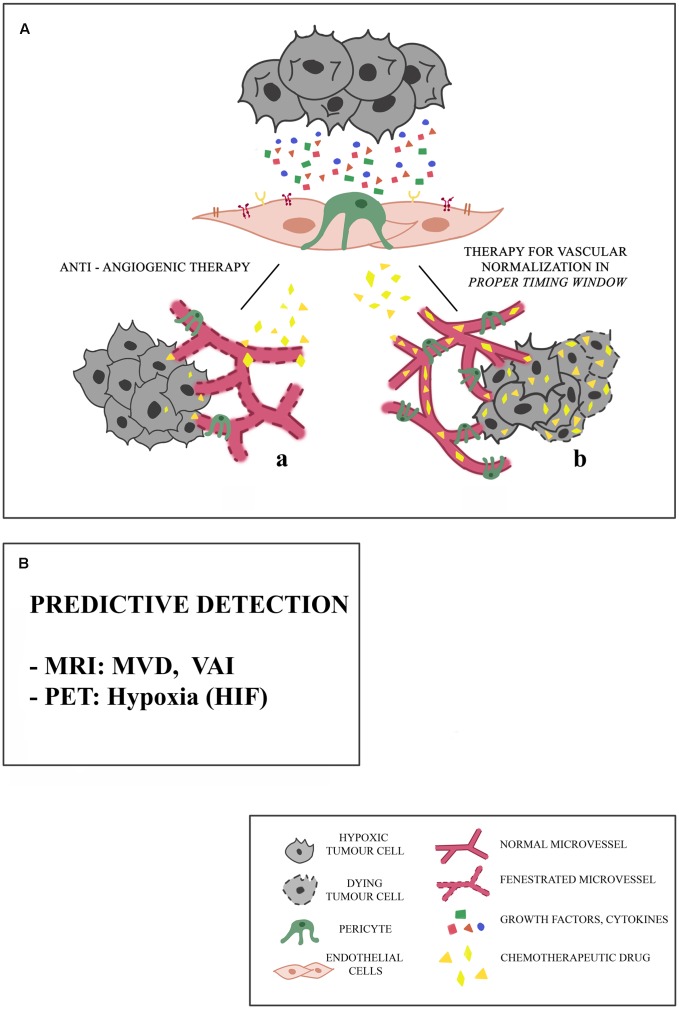
**Proposed mechanisms of microvessel responses to anti-angiogenic therapy. (A)** Tumors may initially respond to anti-angiogenic therapy in different ways, and this response depends on the integrity of microcirculation. **(a)** Because of the presence of fenestrated vessels with a poor pericyte coverage, chemotherapeutic drug cannot reach the targeted tumor site. Consequently, tumor stabilizes or progresses. **(b)** The association of an anti-angiogenic drug with an anti-tumor drug in a *proper timing window* restores a balance between pro- and anti-angiogenic factors, leading to the *normalization* of blood vessels: the chemotherapeutic drug can reach the targeted tumor site. These effects are limited spatially and temporally, and are different in different types of cancers, particularly in the case of little vascularized tumors. **(B)** The predictive detection of microvessel architectural parameters is necessary for the selection of a precision and personal therapy, aimed to the vascular normalization, being these parameters based on Magnetic Resonance Imaging (MRI), Vessel Architectural Imaging (VAI), Microvascular Density (MVD), Positron Emission Tomography (PET).

One of the obstacles to the success of cancer treatment is related to inefficient transport of drugs to cancer cells. Due to lack of proper interconnections between the endothelial cells (ECs), tumor blood vessels are fenestrated and this constitutes a major impediment to the transport and even distribution of the chemotherapy to the tumor tissue (Maes et al., 2016) (**Figure 1A,a**).

Another characteristic of tumor vessels is the lack of pericytes which makes the wall of the vessel thin, changing the permeability within the same tumor and between different tumors (Jain, 2013). The abnormal vascularity can make tumors resistant to chemotherapeutic agents.

The treatment against many types of cancer based on the administration of chemotherapeutic drugs is supported by the use of molecules with anti-angiogenic activity, aimed at reducing tumor blood vessel increase in order to inhibit tumor growth (Teng et al., 2010) (**Figure 1A**).

Phase 3 clinical trials of VEGF pathway inhibitors have shown a significant heterogeneity of tumor response to treatment: tumors can respond to the anti-angiogenic therapy or can give a partial or even no response. A classification of sensitive, partially sensitive and insensitive tumors is reported in **Table 1**.

**Table 1:** Tumor response to anti-angiogenic therapy.

Anti-angiogenic therapy				
**Tumor**	**Sensitive**	**Partially sensitive**	**Insensitive**	**Reference**
Breast cancer		✓		Fakhrejahani and Toi, 2014; Earl et al., 2015
Clear cell renal carcinoma	✓			Hutson et al., 2014; Rini et al., 2014
Colorectal cancer		✓	✓	Bennouna et al., 2013; Grothey et al., 2013
Gastroesophageal cancer		✓		Fuchs et al., 2014; Wilke et al., 2014
Glioma		✓		Gilbert et al., 2014
Hepatocellular carcinoma	✓			Bruix et al., 2015; Cainap et al., 2015
Lung cancer		✓		Garon et al., 2014; Liang et al., 2014
Neuroendocrine and thyroid cancer	✓			Brose et al., 2014
Ovarian and cervical cancer	✓			Pujade-Lauraine et al., 2014; Tewari et al., 2014
Pancreatic cancer			✓	Kindler et al., 2010
Prostate cancer			✓	Michaelson et al., 2014

Moreover, tumor vascularisation may occur *via* alternative mechanisms, which include intussusceptive microvascular growth (Nico et al., 2010), glomeruloid angiogenesis (Straume et al., 2002), looping angiogenesis (Kilarski et al., 2009), vessel co-option and vasculogenic mimicry (Folberg and Maniotis, 2004).

Comparative studies have reported the existence of molecular differences, genetic alterations and drug resistance between normal ECs (NECs) and tumor ECs (TECs). Specific genes for TECs [named tumor endothelial markers (TEMs)] have been shown, and 13 novel cell-surface TEM proteins have been classified (Nanda and Croix, 2004) and are overexpressed during physiological angiogenesis (Seaman et al., 2007). For instance, a VEGF autocrine loop in the first confers resistance to serum starvation, differently from NECs, and TECs are more responsive to VEGF and bFGF than NECs (Matsuda et al., 2010). TECs from highly metastatic tumors show increased sensitivity to VEGF, have less pericyte coverage (Ohga et al., 2012) and disclose the upregulation of angiogenesis-related genes and pathways (Adya et al., 2008). Moreover, tumor cells are able to transdifferentiate into TECs (Wang et al., 2010). Therefore, antineoplastic agents, could not only fail to have access to the tumor mass, but are also relatively active because cells in hypoxia implement mechanisms of resistance (Ebos et al., 2009). All this may explain why a tumor, while being highly vascularised, is often resistant to chemotherapy.

## Anti-Angiogenic Therapies: The Other Side of the Coin

### Tumor Heterogeneity of Response to Anti-angiogenic Therapies

Although the anti-VEGF treatments have constituted a milestone for anti-angiogenic purposes, another aspect of this framework has to be considered in that VEGF inhibitors often fail to produce enduring clinical responses in a great number of patients (Saltz et al., 2007). The anti-angiogenic therapy results in transitory improvements, in the form of tumor standstill or constriction, in some cases increasing survival. In spite of this, tumors begin to grow again, though after a transient period of clinical benefit, generally measured in months (Miller et al., 2005).

Regarding the resistance to the VEGF-targeted therapy, two mechanisms through which this endurance is highlighted can be defined: (i) tumors completely fail to respond from the outset of treatment (*intrinsic resistance*) or (ii) they respond initially, and then continue growing while still receiving the treatment (*acquired resistance*; Bergers and Hanahan, 2008).

### Microvascular Heterogeneity of Response to Anti-angiogenic Therapies

A critical occurrence leading to the success or failure of anti-angiogenic therapy is the need for a *proper timing window* for tumor vascular normalization: long term anti-angiogenic therapy sometimes leads to tumor hypoxia (Winkler et al., 2004), and hypoxia triggers VEGF production, genetic instability in tumor ECs and vascular permeability (Taylor et al., 2010). The decrease in blood flow further reduces oxygen, nutrient and drug delivery, enforcing stress on the tumor (**Figure 1A,a**). In preclinical studies, VEGF-targeted therapy suppresses the growth of new tumor vessels, but is less effective against the established tumor vessels (Sitohy et al., 2012). Nagy and Dvorak (2012) postulated that “early” and “late” tumor vessels might differ in the susceptibility to anti-VEGF therapies. “Early” vessels would predominate initially; the “late” ones become proportionately greater, however, as tumors grow. While the former are responsive, the latter (though formed from the “early” vessels) lose their dependence to the growth factor and become resistant to anti-VEGF-A/VEGFR therapy. Furthermore, the enormous heterogeneity of the tumor vasculature has to be considered, and different types of evolving resistant surrogate tumor blood vessels, which vary between them in anti-VEGF therapy sensitivity, have been described (Sitohy et al., 2012). These aberrant new vessels may be VEGF-independent and therefore capable of mediating tumor vascularisation despite VEGF-inhibition. For example, brain tumors become more infiltrating after VEGF pathway inhibition, which may facilitate vessel co-option (Keunen et al., 2011).

### Cancer Adaptation

Microenvironment adaptation to a cancer stress condition plays a key role in determining whether tumors respond to VEGF-targeted therapies (Rak et al., 2002). This event is driven in part by the molecular promotion of the translation of pro-survival genes, such as BCL2 (Sherrill et al., 2004), X-linked inhibitor of apoptosis protein (XIAP; Gu et al., 2009), and stress response genes (Somers et al., 2015). It has been shown that under the hypoxic condition, which inhibits the global protein synthesis, the eIF4E homolog 4EHP can promote the translation of certain mRNAs involved in the adaptation to hypoxia, such as EGF receptor and PDGF receptor-α (Uniacke et al., 2012). Preclinical data has shown that some adaptive mechanisms include a decreased propensity for certain cancer cells to die under stress conditions, sometimes following genetic aberrations such as loss of p53 function (Yu et al., 2002), by adapting their metabolism (McIntyre et al., 2012) or by autophagy (Xu et al., 2013). In addition to being constituted by transformed cells, tumors are characterized by the presence of infiltrating different stromal cells, which are often the cause of therapy resistance, including the resistance to anti-angiogenic therapies (McMillin et al., 2013). Among these, the immature myeloid cells (Chung et al., 2013), fibroblasts (Crawford et al., 2009) and endothelial progenitor cells (Shaked et al., 2006) infiltrate the tumor and mediate the resistance by incorporating themselves into vessels or by releasing pro-angiogenic growth factors, such as BV8 (Shojaei et al., 2007) or PDGF-C (Crawford et al., 2009). At present, there is conflicting evidence to whether the anti-angiogenic therapy could increase the tumor aggressiveness and cause flare phenomena. In some pre-clinical studies the anti-VEGF treatment promoted this serious downside, in terms of invasion and metastasis (Ebos et al., 2009). Also, evidence in patients showed that anti-VEGF therapy can promote tumor aggression. A study on metastatic renal cancer cell (mRCC) patient demonstrated a significant increase in tumor grade in the primary tumor after treatment with sunitinib and pazopanib (Sharpe et al., 2013), while data from the AVANT trial for colorectal cancer with adjuvant bevacizumab has highlighted that this treatment caused a higher incidence of relapses and deaths (de Gramont et al., 2012). Moreover, anti-VEGF therapy can promote invasion and the undergoing epithelial-to-mesenchymal transition (Lu et al., 2012). In our and other researchers’ opinion, a possible mechanism could be that the anti-angiogenic treatment damages the vessels, causing cancer cell extravasation; in preclinical models, TKIs may promote metastasis by damaging the vasculature integrity (Chung et al., 2012). On the other hand, *flare-up* phenomena have been described in patients with mRCC after the withdrawal of the anti-angiogenic therapy (Powles et al., 2013), and the analysis of the NSABP-C08 trial of adjuvant bevacizumab in colorectal cancer patients did not evidence the noxious effect of bevacizumab (Allegra et al., 2013).

### Evidences of Toxicity

Clinical practices have revealed a large number of adverse complications associated with anti-VEGF treatments. Among these, hypertension, proteinuria, hemorrhage, endocrine dysfunction, thrombosis, gastrointestinal perforation, fistula formation, cardiac toxicity, and reversible posterior leukoencephalopathy (Chen and Cleck, 2009). The need for hypertension treatment has been seen in approximately 25% of patients (Burger et al., 2011). The occurrence of reversible posterior leukoencephalopathy is correlated with uncontrolled hypertension and the permanent cessation of VEGF inhibitor therapy is greatly needed. VEGF inhibitors could also increase the risk of thromboembolism by about 5%, and anticoagulant treatments were active in reducing this side effect. Proteinuria might reflect the hypertension, and VEGF inhibitor treatment is usually stopped when it reaches 3 g protein loss in 24 h (Burger et al., 2011). Although the treatment with bevacizumab (Avastin, which neutralizes specifically the VEGF-A isoforms) and paclitaxel plus carboplatin (both chemotherapeutics) in the treatment of patients with non-small-cell lung cancer had significant survival benefits, febrile neutropenia and pulmonary hemorrhage were associated with the addition of anti-VEGF (Sandler et al., 2006). Moreover, bevacizumab is avoided in patients with ovarian cancer with substantial pelvic disease or with previous bowel surgery for the risk of bowel perforation or fistula formation (Simpkins et al., 2007). Owing to the fact that TKIs inhibit a lot of off-target kinases, they are associated with malaise, fatigue, hypothyroidism, diarrhea, and cardiac failure (Schmidinger and Bellmunt, 2010).

## Need for Pro-Angiogenic Therapy?

Some cancer therapies are based on the association of an anti-angiogenic drug with an anti-tumor drug: the transitional restoration of normal blood vessels allows the drug to reach the tumor site (Jain, 2013). These effects, however, are limited spatially and temporally and are different in different types of cancer and therefore should clearly define the “normalization window” for so the anti-tumor drug can act with maximum efficiency (**Figure 1A,b**).

The approach to the treatment of the cancer with a pro-angiogenic therapy instead of anti-angiogenic, certainly opens up new horizons in therapeutic strategies and leads to a profound change from the clinical point of view. A pro-angiogenic drug can create, around the tumor mass, an extensive network of well-structured blood vessels that facilitate the transport and thus the effectiveness of a cancer drug.

Pericytes are very important in vessel stabilization and maturation, promoting vascular normalization. Data has shown that pericyte loss is a crucial event in early phases of tumor angiogenesis (Anfuso et al., 2014; Lupo et al., 2014; Salmeri et al., 2013). Their presence assure the prevention of metastasis (Xian et al., 2006) and the increment of oxygenation that enhances the sensitization to focal therapies and reduction of tumor growth (Cooke et al., 2012).

Pericyte recruitment is driven by PDGFRβ and its increased expression is a predictor of low survival in breast (Paulsson et al., 2009) and prostate cancers (Hagglof et al., 2010). It has been also demonstrated that PDGF overexpression is associated with increase of melanoma cells proliferation and increased pericyte abundance (Furuhashi et al., 2004). Unfortunately, treatment with imatinib (TKR inhibitor specific to PDGFRβ) has not produced encouraging results in patients with metastatic non-small cell lung cancer (Tsao et al., 2011). Conversely, dual PDGFRβ/VEGFR inhibition is effective for treating multiple stages in tumorigenesis, particularly in solid tumors with high pericyte coverage (Bergers and Hanahan, 2008).

On the other hand, numerous studies have highlighted that the presence of pericytes stabilize tumor microvasculature and allows direct drug delivery to cancer cells (Lo Dico et al., 2015). An increased amount of pericyte on the tumor microvessels inhibits the angiogenesis, while the absence of pericyte coverage correlates with metastasis in colorectal cancer patients (Yonenaga et al., 2005). Particularly noteworthy is the role played by pericytes in response to anti-angiogenic therapy. It has been shown in a pre-clinical study that VEGFR2 blocking is able to determine the recruitment of pericytes, normalizing the tumor microcirculation and allowing the drug to penetrate inside the tumor (Greenberg et al., 2008).

## Predictive Detection

In this scenario, identifying specific parameters, predictive of therapy success, could be necessary for the selection of a targeted anti-tumor drug. These parameters could be provided by imaging techniques (**Figure 1B**).

### Microvascular Density Analyses

Measurements of vessel caliber by Magnetic Resonance Imaging (MRI) is a technique for *in vivo* monitoring of microvascular development during the treatment of cancer patients (Dennie et al., 1998). Some of MRI-based studies, have shown that treatment with anti-angiogenic drugs leads to an improvement in tumor microcirculation, which is less permeable and has an increased pericyte coverage (Goel et al., 2011). By using this technique, it is possible to obtain images of the structure of the tumor microvasculature (Vessel Architectural Imaging, VAI) whose evaluation provides an efficient parameter for monitoring disease progression and response to treatment in cancer patients. Emblem et al. (2013) conducted a retrospective analysis of 30 patients affected by glioblastoma and studied the structural heterogeneity of the tumor microcirculation by VAI, demonstrating that, during the anti-angiogenic therapy, tumor blood vessels of subjects who responded to treatment were similar to those of normal tissues (Sikov et al., 2015). Recent studies have shown a significant increase in the number of patients with breast cancer who are HER2-negative or triple negative and do not respond to conventional anti-angiogenic therapies (Earl et al., 2015), and that an improvement of vascular conditions causes an increase in tumor oxygenation and a better response to anti-angiogenic agents (Heist et al., 2015).

Tolaney et al. (2015) have demonstrated that high baseline microvascular density (MVD) in breast tumors is considered a positive response to vascular normalization index induced by bevacizumab. MVD is calculated by evaluating specific parameters including pericytes coverage and the number of α-SMA. In the case of high MVD, the effect of anti-angiogenic drugs would be to remove some vessels and increase the functions of others, by inducing their normalization. In the case of low MVD, the anti-angiogenic drug reduces them further and prevents their normalization. This makes some tumors insensitive to anti-angiogenic therapy. Consequently, knowing the baseline MVD is a key factor in predicting the success of treatment with anti-angiogenic drugs (Jeong et al., 2015).

### Hypoxia Detection

A characteristic of tumor microenvironment is low oxygen tension, caused by an imbalance in oxygen delivery and consumption. At low pO_2_ levels, cells become radioresistant and, as a vicious circle, the irradiation itself, which induces direct vessel damage, stimulates hypoxia with consequent recruitment of immunosuppressive myeloid cells, contributing to tumor resistance (Russell and Brown, 2013). Conversely, it has also been also demonstrated, in a xenograft tumor model, that VEGF is released at the onset of angiogenesis, independent of HIF (Hendriksen et al., 2009). These conflicting results are ascribable to the different origin of the tumors and to the different areas within the same tumor, characterized by chaotic and complex tumor vascular architecture that determines a better or worse oxygen distribution (Hida et al., 2016).

The lactate, produced by tumor glycolytic metabolism, predicts the response to irradiation of human carcinomas (Sattler et al., 2010), an increased risk of metastases (Brizel et al., 2001) and is able to induce angiogenesis (Hirschhaeuser et al., 2011). These findings indicate that the anaerobic metabolism of cancer cells is strongly related to the increased aggressiveness of a tumor and the possibility of measuring its amount is an important predictor.

HIF-1 activity can be silenced through inhibition of epidermal growth factor receptor (EGFR) or topoisomerase-1 or by anthracyclines (Semenza, 2010). Moreover, HIF-1 activity can be inhibited by new drugs which reduces HIF-1 mRNA amounts (Koh et al., 2008) or which provokes HIF-1 degradation (Kim et al., 2006). The treatment with these drugs during radiotherapy amplifies the irradiation effects prompting tumor vasculature destruction and reduction in growth (Harada et al., 2009). Unfortunately, these exciting results did not apply to all types of tumor because several cancers show little or no hypoxia and do not express HIF activation (Moeller and Dewhirst, 2006; Meijer et al., 2012).

Tumor hypoxia represents an important aspect of the tumor microenvironment. Clinical studies using needle-sensors (Eppendorf)^®^ have demonstrated that hypoxia varied on a tumor-to-tumor basis and represents a universal therapy resistance mechanism (Koch and Evans, 2015). For this reason, several methods have been developed to assess tumor hypoxia and to predict treatment outcome by evaluating the oxygenation status during therapy.

Several studies have been conducted to determine the presence of HIF inside tumor cells. Recent advances in imaging of hypoxia by positron emission tomography (PET) demonstrated that it is possible to select patients for specific therapies, improving the anti-hypoxia-direct radiotherapy (Baumann et al., 2016). Activation of HIF transcription factor can be also evaluated by using genetically encoded fluorescent sensors with different switching and their combination allows the distinction of hypoxic and re-oxygenated cells in glioma cell lines, focusing on regions devoid of blood vessels (Erapaneedi et al., 2016). A potential tracer, used as a biomarker in the context of anti-angiogenic therapy, is [^18^F]-FMISO: a low [^18^F]-FMISO-PET signal is correlated to decreased hypoxia and it is a predictor of vascular normalization (Hernandez-Agudo et al., 2016).

The dynamic contrast enhanced magnetic resonance imaging (DCE-MRI) technique has been used to evaluate the effect of bortezomib, by using multiple endogenous and exogenous markers to evaluate hypoxia (Sun et al., 2014). By DCE-MRI it has been demonstrated that tumor blood flow is significantly reduced after bortezomib administration and the results of this study are very important to monitoring the effects of treatment with an anti-tumoral drug.

It has been recently reported that a hypoxia visualization bio-imaging probe, protein transduction domain [PTD]-oxygen dependent degradation domain [ODD]-HaloTag (POH), was able to detect HIF-1 active (Takata et al., 2015).

HIF activity has also been monitored in a preclinical glioma model. After treatment with different drugs, imaging biomarkers through luciferase expression have been used to document the tumor response (Lo Dico et al., 2015).

## Conclusion

The authors believe that these studies show alternative therapeutic pathways, capable of inducing the differentiation and maturation of tumor blood vessels. In our opinion, the recruitment of pericytes must be taken into account for new strategies in the fight against those tumors, which are especially drug-resistant to traditional therapies.

These studies on new target tracers represent a useful tool for theranostic procedures.

## Author Contributions

CA and GL designed the study. NC, MO, and MC collected the data reported in **Table 1**. CA and GL wrote the manuscript and drew the **Figure 1**. FN, RA, VB, CM, and MS added the helpful discussions. CA, GL, and FN edited the manuscript, figure and table.

## Conflict of Interest Statement

The authors declare that the research was conducted in the absence of any commercial or financial relationships that could be construed as a potential conflict of interest.
